# Inhibition of Notch pathway enhances the anti-tumor effect of docetaxel in prostate cancer stem-like cells

**DOI:** 10.1186/s13287-020-01773-w

**Published:** 2020-06-26

**Authors:** Lei Wang, Hao Zi, Yi Luo, Tongzu Liu, Hang Zheng, Conghua Xie, Xinghuan Wang, Xing Huang

**Affiliations:** 1 Department of Radiation and Medical Oncology, Zhongnan Hospital of Wuhan University, Wuhan, 430071 China; 2 Hubei Key Laboratory of Tumor Biological Behaviors, Wuhan, 430071 China; 3 Hubei Cancer Clinical Study Center, Wuhan, 430071 China; 4 Center for Evidence-Based and Translational Medicine, Zhongnan Hospital of Wuhan University, Wuhan, 430071 China; 5 Department of Urology, Zhongnan Hospital of Wuhan University, Wuhan, 430071 China

**Keywords:** Prostate cancer, Cancer stem cells, Notch, γ-Secretase inhibitor, Docetaxel resistance

## Abstract

**Background:**

Prostate cancer stem-like cells (PCSCs) likely participate in tumor progression and recurrence and demonstrate resistance to chemotherapy. The Notch pathway plays a role in the maintenance of the stemness in PCSCs. This study aimed to investigate the efficacy of Notch signaling inhibition as an adjuvant to docetaxel (DOX) in PCSCs.

**Methods:**

PCSCs derived from the PC-3 cell line were examined for Notch-1 expression. The effect of Notch inhibition on response to DOX was evaluated in PCSCs in vitro and in murine models using a γ-secretase inhibitor (GSI), PF-03084014. Impacts on cell proliferation, apoptosis, cell cycle, and sphere formation were evaluated.

**Results:**

PC-3 PCSCs expressed elevated Notch-1 mRNA compared with PC-3 parental cells. The combination of GSI with DOX promoted DOX-induced cell growth inhibition, apoptosis, cell cycle arrest, and sphere formation in PCSCs. In nude mice bearing PC-3 PCSC-derived tumors, the combination of GSI and DOX reduced the tumor growth, which was associated with the decreased Notch-1 expression in tumor tissues.

**Conclusions:**

These results reveal that inhibition of the Notch pathway enhances the anti-tumor effect of DOX in PC-3 PCSCs, and suggest that Notch inhibition may have clinical benefits in targeting PCSCs.

## Introduction

Prostate cancer is the most common cancer in the USA, and it is estimated that there will be 191,930 newly diagnosed prostate cancer cases and 33,330 deaths due to prostate cancer in 2020 [1]. Conventional therapies, such as radical prostatectomy, radiotherapy, and hormone therapy, are effective in the starting phase of prostate cancer; however, it will eventually progress to metastatic, drug- and castration-resistant prostate cancer [2].

The cancer stem cell (CSC) hypothesis postulates that a small subpopulation of cancer cells drives tumor growth and metastasis and is more resistant to chemo-radiotherapy than differentiated daughter cells [3]. Therefore, CSCs may survive due to their high resistance to drugs which leads to treatment failure [4]. Prostate cancer stem-like cells (PCSCs) have been isolated from human prostate cancer biopsies and cell lines, and they are resistant to chemotherapy and self-renew in vitro [5, 6]. Although docetaxel (DOX) is frequently utilized as a first-line chemotherapy in metastatic and advanced prostate cancer, not all the patients respond to DOX, and in those that do, DOX resistance eventually develops [7, 8]. Thus, targeting PCSCs and enhancing DOX activity would provide a major benefit to patients.

The Notch pathway plays a key role in proliferation, stem cell maintenance, cell fate determination, and differentiation. Moreover, the Notch signaling pathway is vital to tumorigenicity of the CSCs [9]. The inhibition of the Notch pathway may contribute to the therapeutic strategy to cure cancer by eliminating the CSCs [10]. Domingo-Domenech and colleagues identified a subpopulation of prostate cancer cells that are resistant to DOX overexpress Notch and Hedgehog signaling pathways and possess tumor-initiating capacity, and the DOX-resistant cells could be depleted by targeting these pathways [11].

Recently, Cui and colleagues have shown that inhibition of Notch signaling by γ-secretase inhibitor (GSI), PF-03084014, enhances the anti-tumor effect of DOX in prostate cancer [12]. However, it is not completely clear that pharmacological targeting of the Notch pathway could impact DOX chemoresistance in prostate CSCs. We have isolated PCSCs from prostate cancer cell lines and proved that PCSCs have the characteristics of stem cells in our previous study [6]. In this study, we sought to further investigate the efficacy of Notch signaling inhibition as an adjuvant to DOX in PCSCs using GSI (PF-03084014), which might have clinical benefits in targeting PCSCs.

## Methods

### Cell culture and reagents

Human prostate cancer cell line PC-3 was obtained from the American Type Culture Collection (ATCC). The cells were cultured in RPMI-1640 supplemented with 10% heat-inactivated fetal bovine serum (FBS, Gibco/Invitrogen, Australia) and 1% penicillin/streptomycin (Invitrogen) at 37 °C in a humidified incubator in the presence of 5% CO_2_. Docetaxel (DOX) injection and GSI (PF-03084014) were purchased from Sigma-Aldrich (cat. No 01885) and Selleckchem (cat. No S8018), respectively.

### PCSCs

PCSCs have been enriched from the PC-3 cell line as previously described [6]. PCSCs were cultured in EF20 medium composed of neurobasal medium (Invitrogen) supplemented with 3 mM l-glutamine (Mediatech), 20 ng/mL human EGF (R&D Systems), 20 ng/mL human FGF-2 (PeproTech), 1 × B27 supplement (Invitrogen), 0.5 × N2 supplement (Invitrogen), 2 mg/mL heparin (Sigma), and 0.5 × penicillin G-streptomycin sulfate-amphotericin B complex (Mediatech). For passaging, spheres were centrifuged, treated with Accutase (Innovative Cell Technologies) at 37 °C for 5 min, dissociated by pipetting, washed with PBS, and resuspended in EF20 medium [13].

### Quantitative real-time PCR

Total RNA was extracted from PC-3 parental cells and PCSCs using TRIzol Plus RNA Purification Kit (Invitrogen) according to the manufacturer’s protocol. Using RevertAid First Strand cDNA Synthesis Kit (Fermentas), mRNA was reverse-transcribed into cDNA. Quantitative real-time reverse transcriptase polymerase chain reaction (qRT-PCR) was performed using the SYBR Green Master Mix (Fermentas). The primers were as follows: Notch-1 (forward 5′-CCTGTCTGAGGTCAATGAGT-3′; reverse 5′-GTAGCCACTGGTCATGTCTT-3′); Oct-4 (forward 5′-CGAAAGAGAAAGCGAACCAG-3′; reverse 5′-GCCGGTTACAGAACCACACT-3′); Nanog (forward 5′-AAGGTCCCGGTCAAGAAACAG-3′; reverse 5′-CTTCTGCGTCACACCATTGC-3′); GAPDH (forward 5′- AGAAGGCTGGGGCTCATTTG-3′; reverse 5′-AGGGGCCATCCACAGTCTTC-3′). In order to compare the relative expression of the mRNA in different samples, the comparative delta Ct (threshold cycle number) was calculated.

### Cell susceptibility assay

PC-3 parental cells and PCSCs were seeded into 96-well plates at 3000 cells/well for overnight. Cells were treated for 72 h with DOX (0.001, 0.01, 0.1, 1, 10, and 100 nM), or GSI (0.01, 0.03, 0.1, 0.3, 1, 3, 10, and 30 μM), or DOX (0.001, 0.01, 0.1, 1, 10, and 100 nM) and GSI (5 μM). 5-(3-Carboxymethoxyphenyl)-2-(4,5-dimethylthiazoly)-3-(4-sulfophenyl) tetrazolium, inner salt (MTS) assays (Promega) were performed according to the manufacturer’s instructions, and dose-response curves were plotted (non-linear fit; GraphPad Prism).

### Flow cytometric analysis

Flow cytometric analysis was used to detect the apoptosis (using FITC Annexin V Apoptosis Detection Kit, BD Pharmingen™) and cell cycle distribution (using Propidium Iodide Staining, Sigma) of PC-3 PCSCs treated with (1) vehicle (control, DMSO), (2) DOX (10 nM), (3) GSI (5 μM), and (4) DOX (10 nM) + GSI (5 μM) for 72 h. Flow cytometric analysis was performed using a Beckman Coulter FC 500 MCL/MPL counter fitted with a 488-nm laser.

### Sphere formation assay

PC-3 PCSCs were dissociated and passed through a 100-μm filter to produce single-cell suspensions. One thousand cells were cultured in 100 μL EF20 media/well in 96-well plates and treated with (1) vehicle (control, DMSO), (2) DOX (10 nM), (3) GSI (5 μM), and (4) DOX (10 nM) + GSI (5 μM). Then, 14 days later, sphere (diameters ≥ 50 μm) size and numbers in each well were measured using microscopy. Sphere formation ratio (%) was calculated using the following formula: sphere formation ratio (%) = (numbers of wells with spheres) ÷ (total wells seeded with PCSCs) × 100.

### In vivo studies

Six- to 8-week-old male BALB/c nude mice were purchased from Hunan SLK Laboratory Animal Center (Hunan, China) and housed under standard conditions. After 1 week of adaptation, the animals were used for in vivo studies. All experimental protocols were approved by the Animal Care Committee of Wuhan University. PC-3 PCSCs (5 × 10^4^ in 100 μL) were injected subcutaneously into the flank of the mice. Tumor volume (*V*) was calculated following the formula: *V* (mm^3^) = *a*^2^ × *b* × 0.52, where *a* and *b* are the shortest and longest diameters, respectively. Tumor growth was measured using Vernier calipers. Three to 4 weeks after inoculation, when the tumor volumes were approximately 100–200 mm^3^, the mice were randomly assigned to different treatment groups (7 mice per group): (1) control (DMSO), (2) DOX (10 mg/kg, i.p., weekly for 4 weeks), (3) GSI (150 mg/kg, daily, p.o., 7-days-on/7-days-off schedule for 4 weeks based on a previous report [14]), and (4) DOX + GSI (same dosage as above for 4 weeks). There was no significant difference in mean tumor volumes and body weight across all groups at the beginning of treatment. Tumor size and body weight were measured twice a week. At the end of treatment, tumor specimens were fixed in 4% paraformaldehyde, embedded in paraffin, and cut into 5-μm-thick slides. The expression of Notch-1 (1:150, Santa Cruz Biotechnology) was evaluated by immunofluorescent staining according to the manufacturer’s instructions. Sections were examined for positive staining that was quantified as previously described [13].

### Statistical analysis

All experiments were repeated at least in triplicate. Collected data were analyzed using GraphPad Prism 5.0 software (GraphPad Software, Inc., San Diego, CA), and results were displayed as mean ± SD. Comparison between two groups was analyzed with unpaired Student’s *t* test (two tailed), and differences among more than two groups were determined by a one-way ANOVA followed by Newman-Keuls test. Difference with *p* < 0.05 was considered statistically significant.

## Results

### Notch-1 receptor expression is increased in PC-3 PCSCs

**Fig. 1:**
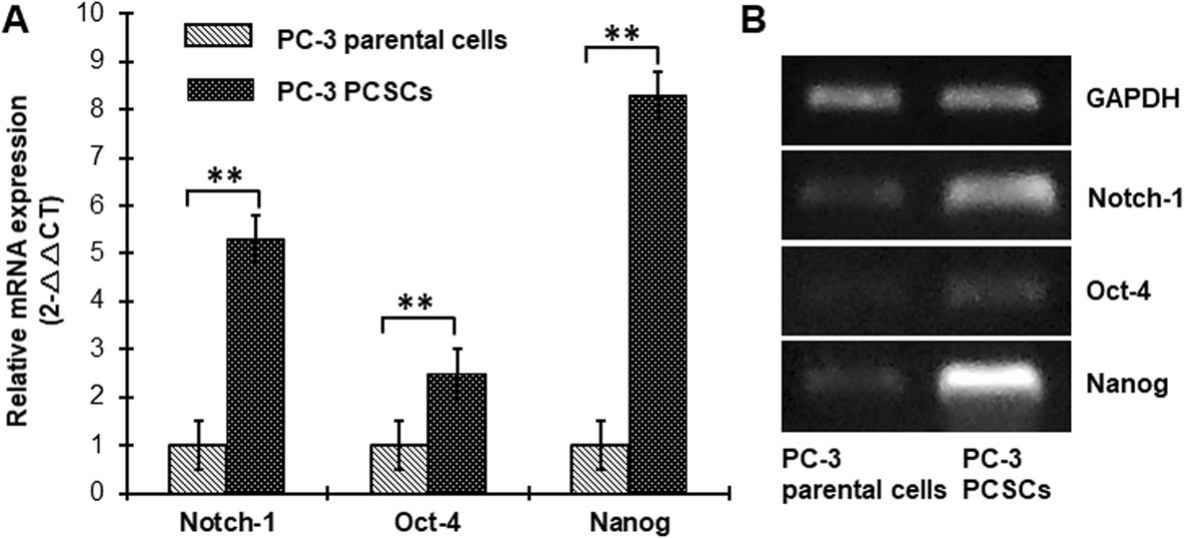
Notch-1 receptor expression is increased in PC-3 PCSCs. **a** qRT-PCR analysis of Notch-1, Oct-4, and Nanog mRNA expression in PC-3 PCSCs and parental cells. **b** The mRNA levels of Notch-1, Oct-4, and Nanog in PC-3 PCSCs and parental cells. **p < 0.01

We cultured human PC-3 prostate cancer cells in growth factor defined (epidermal growth factor [EGF] and fibroblast growth factor [FGF]-2) serum-free medium for the enrichment of PCSCs and detected the stem cell markers including Notch-1, Oct-4, and Nanog in PCSCs and parental cells. After enrichment of PC-3 PCSCs, the expression of Notch-1, Oct-4, and Nanog mRNA were significantly increased in PCSCs, by 5.3-, 2.5-, and 8.3-fold, respectively, compared to parental cells (Fig. 1).

### GSI enhances the chemosensitivity of PC-3 PCSCs to DOX

**Fig. 2:**
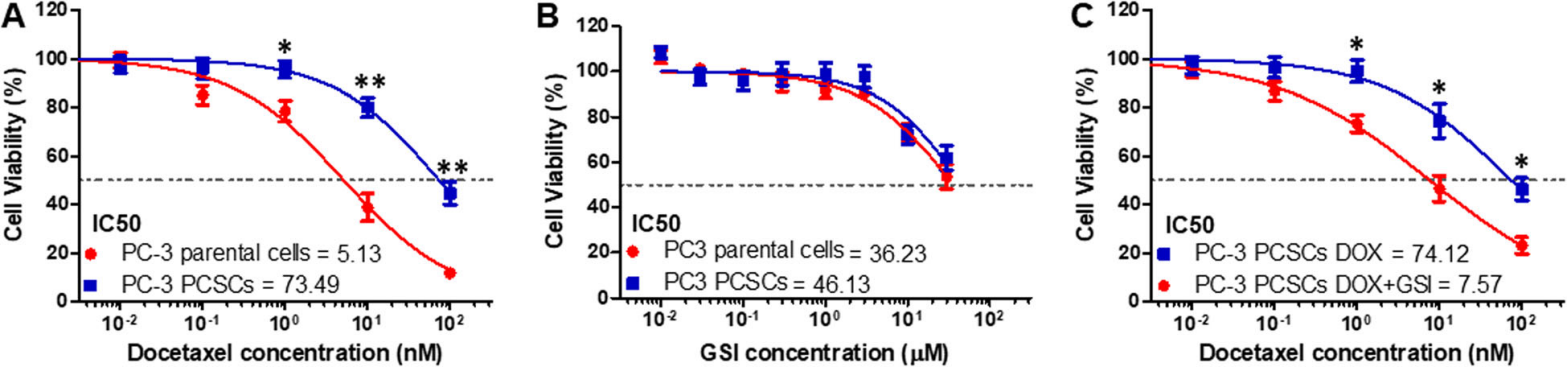
GSI enhances the chemosensitivity of PC-3 PCSCs to DOX. Cell viability of PC-3 parental cells and PCSCs treated with DOX (**a**), GSI (**b**), or the combination of DOX with GSI (**c**) for 72 h. Data are shown as mean ± SD. *p < 0.05, **p < 0.01

CSCs have been shown to exhibit resistance to chemotherapy [3]. We examined whether PCSCs have a differential response to DOX compared with parental cells. PC-3 PCSCs were more chemoresistant than PC-3 parental cells (Fig. 2a). To determine whether GSI has different effects on the proliferation of PC-3 parental cells and PCSCs, we evaluated the viability of PC-3 parental cells and PCSCs treated with GSI. PC-3 PCSCs had similar sensitivity to GSI as parental cells (Fig. 2b). Then, we evaluated the effect of GSI on responsiveness to DOX in PC-3 PCSCs. The inhibition of cell proliferation by the combination of DOX and GSI was significantly higher than that observed in PC-3 PCSCs treated with DOX alone (Fig. 2c).

### GSI promotes apoptosis and cell cycle arrest induced by DOX in PC-3 PCSCs

**Fig. 3:**
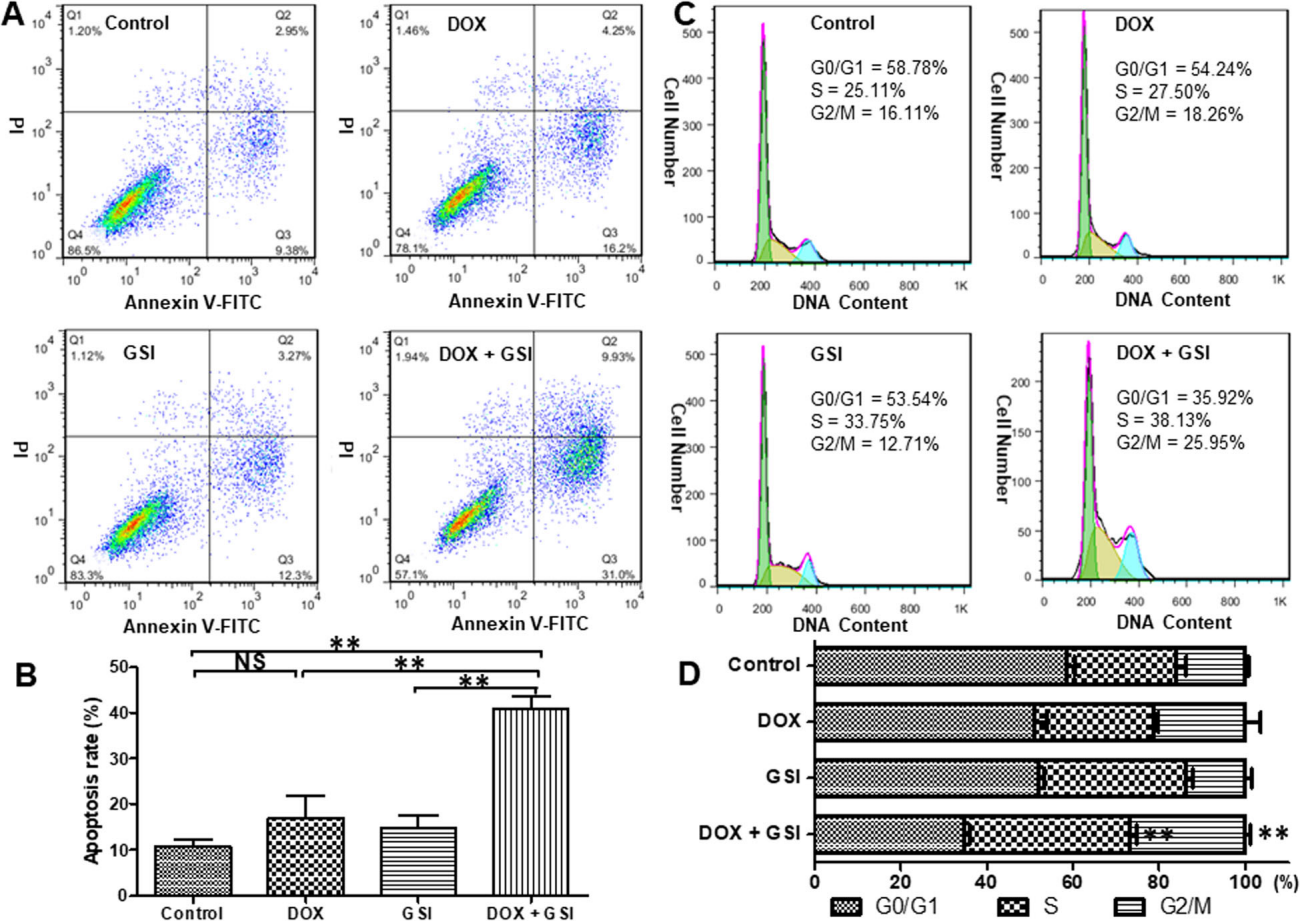
GSI promotes apoptosis and cell cycle arrest induced by DOX in PC-3 PCSCs. **a** The apoptosis rate of PC-3 PCSCs treated with vehicle, DOX, GSI, or the combination of DOX and GSI was detected using flow cytometry. Flow charts: lower right quadrant, annexin V-positive and PI-negative cells indicate early apoptotic cells; upper right quadrant, annexin V- and PI-positive cells represent necrotic or late apoptotic cells. Both early and late apoptotic cells were calculated as the incidence of apoptotic cell death. **b** The statistical data of the apoptosis rates under different conditions for 72 h. **c** Cell cycle distribution of PC-3 PCSCs treated with the combination of DOX and GSI or each one alone was detected by flow cytometry. **d** The statistical data of cell cycle distribution under different conditions for 72 h. **p < 0.01, NS no significant difference

DOX is a taxane antimitotic agent used as the standard chemotherapy in metastatic and advanced prostate cancer. The impacts on apoptosis and cell cycle distribution of PC-3 PCSCs treated with the combination of DOX and GSI or each one alone were detected by flow cytometry. The results showed that the apoptosis rate was significantly increased in PC-3 PCSCs treated with the combination of DOX and GSI compared to single agent treatment (Fig. 3a, b). Furthermore, the combination of DOX and GSI led to a significant arrest in cell cycle progression, as the number of cells in the S and G2/M phases was markedly increased (Fig. 3c, d).

### Combination therapy inhibits the self-renewal potentiality of PC-3 PCSCs

**Fig. 4:**
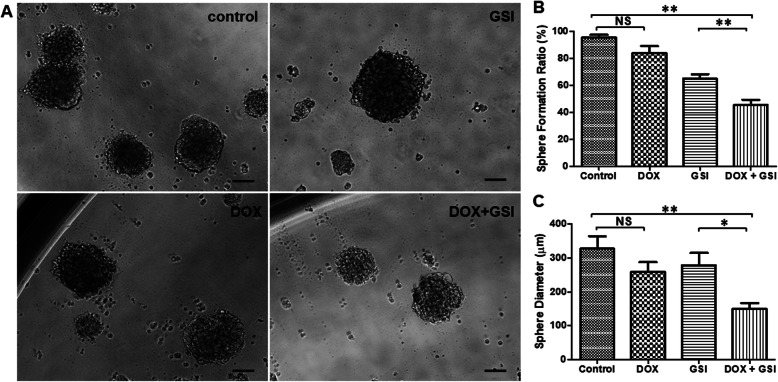
Combination therapy inhibits the self-renewal potentiality of PC-3 PCSCs. **a** Morphology of spheres derived from different treatments of vehicle (control), DOX, GSI, or the combination of DOX with GSI in 1000 cells/well of PC-3 PCSCs (*n* = 60 wells). **b** The statistical data of sphere formation ratio in PC-3 PCSCs under different conditions. **c** The statistical data of sphere diameters in PC-3 PCSCs under different conditions. *p < 0.05, **p < 0.01, NS no significant difference. Scale bars, 100 μm

CSCs have self-renewal capacity and are considered linked to elevated Notch pathway signal. PC-3 PCSCs were resistant to chemotherapy, and DOX treatment did not inhibit the sphere formation of PC-3 PCSCs (Fig. 4a, b). However, inhibition of the Notch pathway by GSI decreased the sphere formation, and the combination treatment with DOX and GSI induced a significant decrease in sphere formation of PC-3 PCSCs compared to GSI treatment alone (Fig. 4a, b). In addition, the combination therapy resulted in smaller sphere size than that in monotherapy groups (Fig. 4c). The sphere formation results suggested a synergistic effect of DOX and GSI in inhibiting self-renew of PC-3 PCSCs.

### Combination of GSI with DOX reduces the tumor growth in vivo

**Fig. 5:**
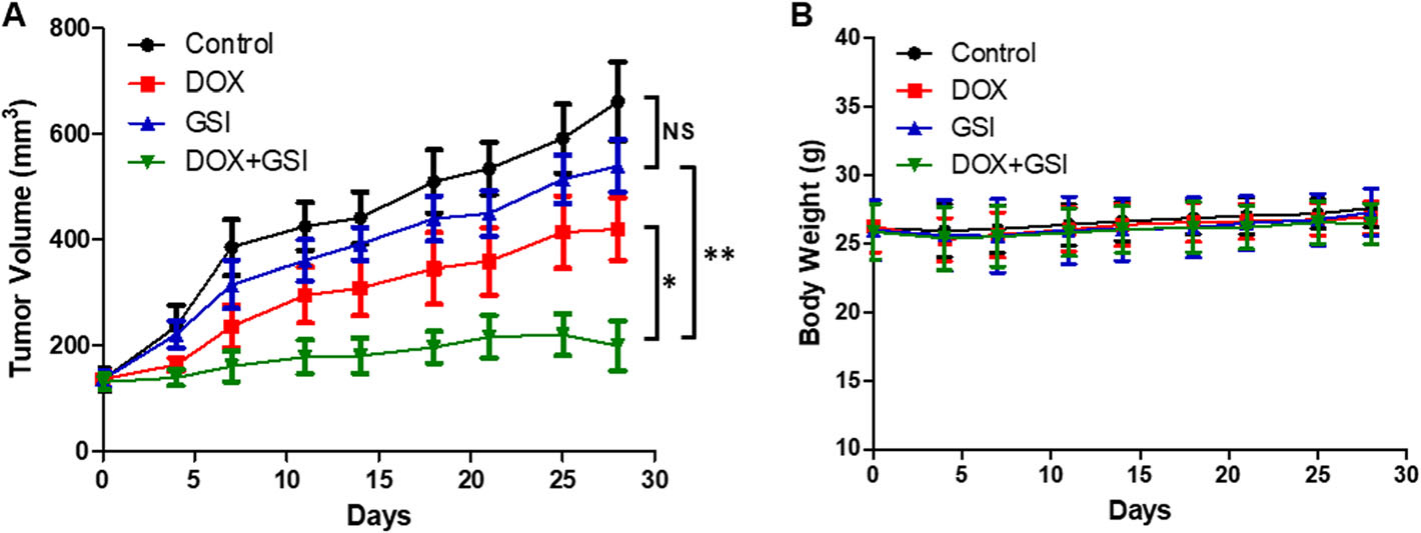
The combination of GSI with DOX reduces PC-3 PCSC-derived tumor growth in vivo*.***a** Tumor volume of PC-3 PCSC xenografts in BALB/c nude mice treated with vehicle (control), DOX, GSI, or the combination of DOX with GSI. **b** Body weight over time of nude mice bearing PC-3 PCSC tumor xenografts for control and treatment groups. *p < 0.05, **p < 0.01, NS no significant difference

**Fig. 6:**
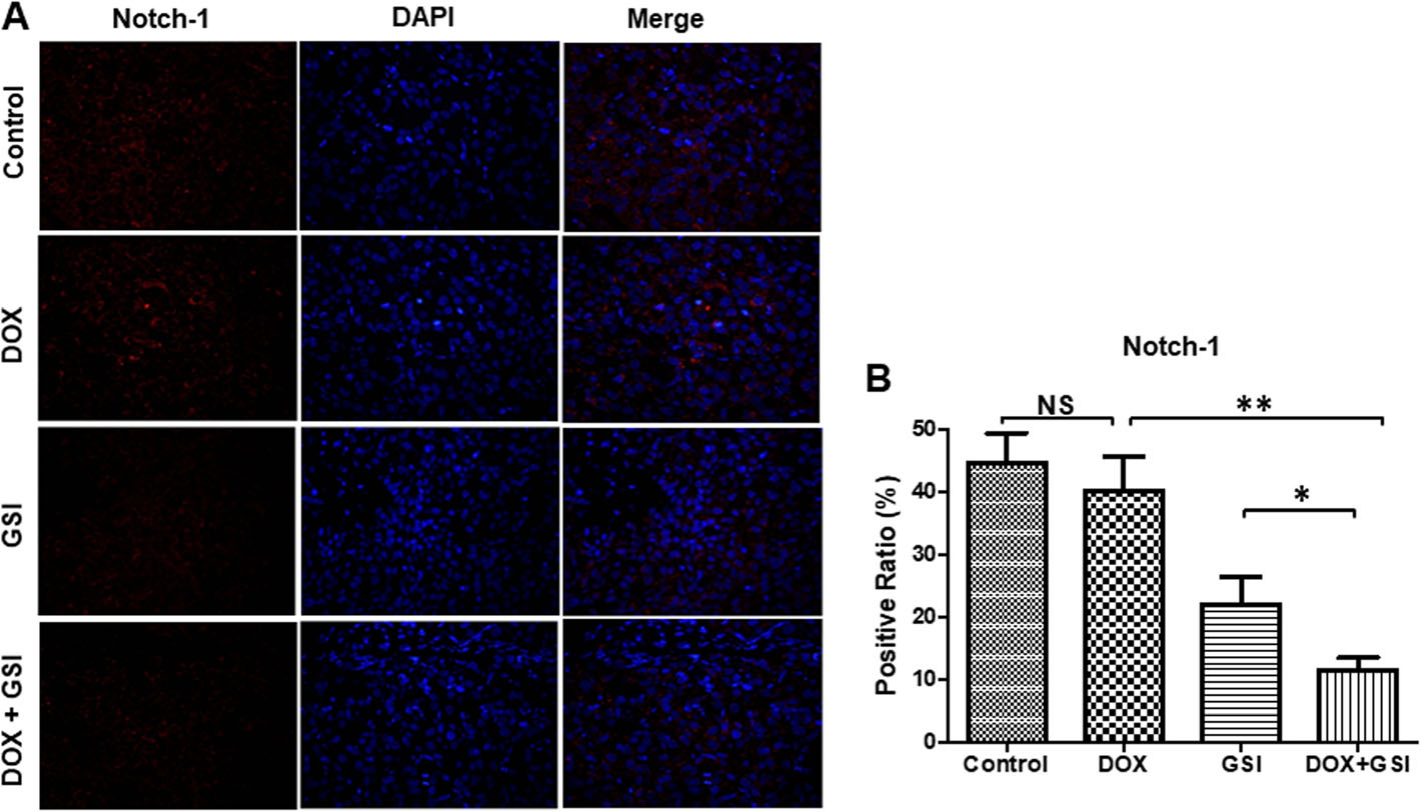
The combination therapy decreases the Notch-1 expression in PC-3 PCSC-derived tumor tissues. **a** Immunofluorescent staining (× 200) of Notch-1 (red) and DAPI (blue) in PC-3 PCSC-derived tumor xenografts treated with vehicle (control), DOX, GSI, or the combination of DOX with GSI. **b** Quantitation of Notch-1 expression in PC-3 PCSC-derived tumor xenografts under different conditions. *p < 0.05, **p < 0.01, NS no significant difference

We evaluated the efficacy of monotherapy and combination therapy in subcutaneous PC-3 PCSC-derived tumors. At the end of the 4-week treatment schedule, there was no significant difference in tumor volume between control and GSI treatment groups. However, the combination therapy of DOX with GSI had synergistic growth inhibitory effects when compared with DOX or GSI alone (Fig. 5a). There were no significant decreases in the mean body weights of mice treated with DOX, GSI, or combination therapy compared with the control group (Fig. 5b), which indicates no severe toxicity due to monotherapy or combination therapy. We next explored the expression of Notch-1 in the tumor tissues among these groups. The percentage of Notch-1-positive cells were 44.7%, 40.2%, 22.0%, and 11.5% respectively in control, DOX, GSI, and combination therapy groups. The results showed that combination of DOX with GSI significantly decreased the Notch-1 expression in the tumor tissues when compared with DOX or GSI alone (Fig. 6).

## Discussion

Recently, emerging evidence suggests that PCSCs may play a pivotal role in the development and progression of prostate cancer [15, 16]. The Notch signaling pathway is vital to tumorigenicity of the CSCs, and thus receiving increased attention as a target to eliminate CSCs [9, 10]. Therefore, targeting PCSCs through inhibition of the Notch pathway could become a novel strategy for better treatment of patients diagnosed with prostate cancer. PCSCs have been isolated from the PC-3 cell line and demonstrate resistance to chemotherapy in our previous study [6]. In the present study, we found that the expression of Notch-1 was increased in PC-3 PCSCs compared to parental cells. Furthermore, inhibition of the Notch pathway using GSI enhanced the anti-tumor effect of DOX in PC-3 PCSCs. Thus, targeting PCSCs and enhancing DOX activity would provide a major benefit to patients with advanced prostate cancer.

The Notch signaling pathway controls cell fate decisions during development, including differentiation, proliferation, stem cell maintenance, and self-renewal of various cell types [17]. Moreover, increasing evidence has shown that the Notch pathway critically regulates the self-renewal and survival of CSCs in breast cancer, embryonal brain tumors, and gliomas [18]. In terms of prostate cancer, Oktem and colleagues identified CD133^high^/CD44^high^ DU145 prostate CSCs and found that Jagged1, Delta-like 3, and Notch-1 were respectively upregulated genes in the Notch signaling pathway appearing to be due to malignancy and tumor progression [19]. Furthermore, Liu and colleagues revealed that the protein level of activated form of Notch-1 was significantly higher in PCSCs isolated from LNCaP and PC-3 cell lines, and inhibition of Notch-1 with shRNA could improve chemosensitivity in PCSCs [20]. We have proved that PCSCs are resistant to DOX in our previous study [6]. In the present study, we also found the expression of Notch-1 mRNA was significantly increased in PC-3 PCSCs compared to parental cells, and inhibition of the Notch pathway by GSI can enhance the chemosensitivity of PCSCs to DOX. These findings suggest that the Notch pathway may play a key role in the tumorigenicity and chemoresistant of PCSCs. Therefore, targeting Notch signaling pathway as a therapeutic strategy for treating cancer has attracted increasing interest.

γ-Secretase is a key mediator of Notch signaling [21]. The γ-secretase enzyme activity that helps in the proteolytic cleavage of the receptors results in the release of the Notch intracellular domain and is therefore a central player in the Notch signaling pathway [10]. Thus, GSI is a promising target for Notch inhibition. PF-03084014 is a selective reversible, non-competitive GSI that inhibits the Notch pathway through blocking proteolytic activation of Notch receptors in various types of cancer [14, 22–24]. Recently, a few studies have found that inhibition of Notch signaling using PF-03084014 sensitized DOX-resistant prostate cancer cells to DOX [12, 25]. However, it is not completely clear that pharmacological targeting of the Notch pathway by PF-03084014 could impact DOX chemoresistance in prostate CSCs. In this study, we revealed that PF-03084014 enhanced the chemosensitivity of PC-3 PCSCs to DOX, promoted apoptosis and cell cycle arrest induced by DOX, and inhibited the self-renewal potentiality of PC-3 PCSCs in vitro. In addition, combination therapy of GSI with DOX reduced the PC-3 PCSC-derived tumor growth in vivo, which was associated with the decreased Notch-1 expression in tumor tissues.

DOX is an antimitotic chemotherapy drug that interferes with cell division and is widely used for the treatment of multiple types of cancer. However, recent studies have suggested CSCs as a main player for chemoresistance against a variety of drugs including DOX [26]. In our study, PF-03084014 enhanced the chemosensitivity and promoted apoptosis of PC-3 PCSCs to DOX due to the suppression of PCSCs by the inhibition of Notch pathway. Alessio and colleagues found that multilineage-differentiating stress enduring (Muse) cells were resistant to chemical and physical genotoxic stresses better than non-Muse cells, and the level of senescence and apoptosis was lower, which is related to their quick and efficient sensing of DNA damage and activation of DNA repair systems [27]. However, it is unclear whether CSCs will be prone to apoptosis or senescence after chemical and physical genotoxic stresses, so it needs further investigation. In prostate cancer cells, DOX caused phosphorylation and hence inactivation of CDC2 kinase resulting in G2/M arrest [28], whereas targeting the Notch pathway induced cell cycle arrest at S phase [29]. The combination of DOX and GSI induced S and G2/M arrest in PC-3 PCSCs, suggesting that a combination of the two agents may complement each other to result in enhanced arrest.

CSCs have self-renewal capacity and are considered linked to elevated Notch pathway signal [18]. The sphere formation assay is a classic method for the analysis of their self-renewal ability [3]. PC-3 PCSCs were resistant to chemotherapy, and DOX treatment did not inhibit the sphere formation of PC-3 PCSCs. However, the combination of DOX with GSI decreased the sphere formation and resulted in smaller sphere size in PC-3 PCSCs compared with monotherapy. The sphere formation results suggested a synergistic effect of DOX and GSI in inhibiting self-renew of PC-3 PCSCs.

The well-known toxicities of GSI are associated with the gastrointestinal tract leading to severe adverse diarrhea [10]. The intermittent dosing of PF-03084014 reduced gastrointestinal toxicity [14]. Thereby, we used the intermittent dosage regimen of PF-03084014 in the animal study, and there were no significant decreases in the mean body weights of mice treated with DOX, GSI, or combination therapy compared with the control group, which indicates no severe toxicity due to monotherapy or combination therapy. Thus, DOX combined with GSI was not only effective but safe and well tolerated.

However, it should be recognized that tissue-specific stem cell therapy for prostate cancer is still in its infant stage and much more studies and optimizations are required to execute its full power in clinical treatment of prostate cancer patients [15]. Therefore, the specific mechanism of combination therapy of DOX with GSI on PCSCs needs further investigation, and this combination therapy should be evaluated in clinical trials for the therapy of advanced prostate cancer.

## Conclusions

In conclusion, the results presented in this study show that the Notch signaling pathway was upregulated in PC-3 PCSCs. This finding further enabled us to uncover the synergistic effect of a combination of DOX with GSI on PCSCs. We demonstrated that inhibition of the Notch pathway by GSI enhances the anti-tumor effect of DOX in PCSCs, suggesting that Notch inhibition may have clinical benefits in targeting PCSCs. The synergy with GSI suggests that this combination warrants clinical evaluation.

## Data Availability

The datasets supporting the results of this article are included within the article.

## Abbreviations

ATCC: American Type Culture Collection
CSCs: Cancer stem cells
DOX: Docetaxel
FBS: Fetal bovine serum
GAPDH: Glyceraldehyde-3-phosphate dehydrogenase
GSI: γ-Secretase inhibitor
MTS: 5-(3-Carboxymethoxyphenyl)-2-(4,5-dimethylthiazoly)-3-(4-sulfophenyl) tetrazolium, inner salt
PCSCs: Prostate cancer stem-like cells
PI: Propidium iodide
qRT-PCR: Quantitative real-time reverse transcriptase polymerase chain reaction

## References

[1] Siegel RL, Miller KD, Jemal A (2020). Cancer statistics, 2020. CA Cancer J Clin.

[2] Attard G, Parker C, Eeles RA, Schroder F, Tomlins SA, Tannock I, Drake CG, de Bono JS (2016). Prostate cancer. Lancet..

[3] Batlle E, Clevers H (2017). Cancer stem cells revisited. Nat Med.

[4] Giordano A, Fucito A, Romano G, Marino IR (2007). Carcinogenesis and environment: the cancer stem cell hypothesis and implications for the development of novel therapeutics and diagnostics. Front Biosci.

[5] Collins AT, Berry PA, Hyde C, Stower MJ, Maitland NJ (2005). Prospective identification of tumorigenic prostate cancer stem cells. Cancer Res.

[6] Wang L, Huang X, Zheng X, Wang X, Li S, Zhang L, Yang Z, Xia Z (2013). Enrichment of prostate cancer stem-like cells from human prostate cancer cell lines by culture in serum-free medium and chemoradiotherapy. Int J Biol Sci.

[7] Tannock IF, de Wit R, Berry WR, Horti J, Pluzanska A, Chi KN, Oudard S, Theodore C, James ND, Turesson I (2004). Docetaxel plus prednisone or mitoxantrone plus prednisone for advanced prostate cancer. N Engl J Med.

[8] Francini E, Petrioli R, Rossi G, Laera L, Roviello G (2014). PSA response rate as a surrogate marker for median overall survival in docetaxel-based first-line treatments for patients with metastatic castration-resistant prostate cancer: an analysis of 22 trials. Tumour Biol.

[9] Takebe N, Miele L, Harris PJ, Jeong W, Bando H, Kahn M, Yang SX, Ivy SP (2015). Targeting Notch, Hedgehog, and Wnt pathways in cancer stem cells: clinical update. Nat Rev Clin Oncol.

[10] Venkatesh V, Nataraj R, Thangaraj GS, Karthikeyan M, Gnanasekaran A, Kaginelli SB, Kuppanna G, Kallappa CG, Basalingappa KM (2018). Targeting Notch signalling pathway of cancer stem cells. Stem Cell Investig.

[11] Domingo-Domenech J, Vidal SJ, Rodriguez-Bravo V, Castillo-Martin M, Quinn SA, Rodriguez-Barrueco R, Bonal DM, Charytonowicz E, Gladoun N, de la Iglesia-Vicente J (2012). Suppression of acquired docetaxel resistance in prostate cancer through depletion of notch- and hedgehog-dependent tumor-initiating cells. Cancer Cell.

[12] Cui D, Dai J, Keller JM, Mizokami A, Xia S, Keller ET (2015). Notch pathway inhibition using PF-03084014, a gamma-secretase inhibitor (GSI), enhances the antitumor effect of docetaxel in prostate cancer. Clin Cancer Res.

[13] Wang L, Ning J, Wakimoto H, Wu S, Wu CL, Humphrey MR, Rabkin SD, Martuza RL (2019). Oncolytic herpes simplex virus and PI3K inhibitor BKM120 synergize to promote killing of prostate cancer stem-like cells. Mol Ther Oncolytics.

[14] Wei P, Walls M, Qiu M, Ding R, Denlinger RH, Wong A, Tsaparikos K, Jani JP, Hosea N, Sands M (2010). Evaluation of selective gamma-secretase inhibitor PF-03084014 for its antitumor efficacy and gastrointestinal safety to guide optimal clinical trial design. Mol Cancer Ther.

[15] Wang G, Wang Z, Sarkar FH, Wei W (2012). Targeting prostate cancer stem cells for cancer therapy. Discov Med.

[16] Rybak AP, Bristow RG, Kapoor A (2015). Prostate cancer stem cells: deciphering the origins and pathways involved in prostate tumorigenesis and aggression. Oncotarget..

[17] LaFoya B, Munroe JA, Mia MM, Detweiler MA, Crow JJ, Wood T, Roth S, Sharma B, Albig AR (2016). Notch: a multi-functional integrating system of microenvironmental signals. Dev Biol.

[18] Pannuti A, Foreman K, Rizzo P, Osipo C, Golde T, Osborne B, Miele L (2010). Targeting Notch to target cancer stem cells. Clin Cancer Res.

[19] Oktem G, Bilir A, Uslu R, Inan SV, Demiray SB, Atmaca H, Ayla S, Sercan O, Uysal A (2014). Expression profiling of stem cell signaling alters with spheroid formation in CD133(high)/CD44(high) prostate cancer stem cells. Oncol Lett.

[20] Liu C, Li Z, Bi L, Li K, Zhou B, Xu C, Huang J, Xu K (2014). NOTCH1 signaling promotes chemoresistance via regulating ABCC1 expression in prostate cancer stem cells. Mol Cell Biochem.

[21] Shih Ie M, Wang TL (2007). Notch signaling, gamma-secretase inhibitors, and cancer therapy. Cancer Res.

[22] Zhang CC, Pavlicek A, Zhang Q, Lira ME, Painter CL, Yan Z, Zheng X, Lee NV, Ozeck M, Qiu M (2012). Biomarker and pharmacologic evaluation of the gamma-secretase inhibitor PF-03084014 in breast cancer models. Clin Cancer Res.

[23] Yabuuchi S, Pai SG, Campbell NR, de Wilde RF, De Oliveira E, Korangath P, Streppel MM, Rasheed ZA, Hidalgo M, Maitra A (2013). Notch signaling pathway targeted therapy suppresses tumor progression and metastatic spread in pancreatic cancer. Cancer Lett.

[24] Arcaroli JJ, Quackenbush KS, Purkey A, Powell RW, Pitts TM, Bagby S, Tan AC, Cross B, McPhillips K, Song EK (2013). Tumours with elevated levels of the Notch and Wnt pathways exhibit efficacy to PF-03084014, a gamma-secretase inhibitor, in a preclinical colorectal explant model. Br J Cancer.

[25] Du Z, Li L, Sun W, Wang X, Zhang Y, Chen Z, Yuan M, Quan Z, Liu N, Hao Y (2018). HepaCAM inhibits the malignant behavior of castration-resistant prostate cancer cells by downregulating Notch signaling and PF-3084014 (a gamma-secretase inhibitor) partly reverses the resistance of refractory prostate cancer to docetaxel and enzalutamide in vitro. Int J Oncol.

[26] Zhao J (2016). Cancer stem cells and chemoresistance: the smartest survives the raid. Pharmacol Ther.

[27] Alessio N, Squillaro T, Özcan S, Di Bernardo G, Venditti M, Melone M, Peluso G, Galderisi U (2018). Stress and stem cells: adult Muse cells tolerate extensive genotoxic stimuli better than mesenchymal stromal cells. Oncotarget..

[28] Nehme A, Varadarajan P, Sellakumar G, Gerhold M, Niedner H, Zhang Q, Lin X, Christen RD (2001). Modulation of docetaxel-induced apoptosis and cell cycle arrest by all-trans retinoic acid in prostate cancer cells. Br J Cancer.

[29] Zhang Y, Wang Z, Ahmed F, Banerjee S, Li Y, Sarkar FH (2006). Down-regulation of Jagged-1 induces cell growth inhibition and S phase arrest in prostate cancer cells. Int J Cancer.

